# Landfill site selection using MCDM methods and GIS in the central part of the Nile Delta, Egypt

**DOI:** 10.1007/s10661-023-11946-8

**Published:** 2023-11-02

**Authors:** Asaad M. Armanuos, Khaled A. Elgaafary, Tamer A. Gado

**Affiliations:** Department of Irrigation and Hydraulics Engineering, Faculty of Engineering, Tanta University, Tanta, Egypt

**Keywords:** Landfill, Nile Delta, Groundwater, Groundwater level, Multi-criteria decision-making, Suitability maps

## Abstract

One of the most prevalent and serious issues afflicting developing countries is the lack of adequate space for waste disposal. Al-Gharbia Governorate, located in the middle of the Nile Delta in Egypt, suffers from random selection of sites for solid waste disposal, resulting in significant environmental challenges. The aim of this study is to determine optimal landfill locations within Al-Gharbia Governorate and validate the existing landfill sites. Four techniques of multi-criteria decision-making (MCDM) were applied to generate suitability maps for the Governorate: the analytical hierarchy procedure (AHP), ratio scale weighting (RSW), straight rank sum (SRS), and Boolean method. Eleven effective criteria were considered: groundwater, surface water, elevation, slope, soils, land use, roads, railways, urban areas, villages, and power lines. The suitability maps were categorized into four different classes: suitable, moderately suitable, low suitable, and unsuitable. The latest suitability map was determined by combining the results from the different methods, providing decision-makers with the means to select the optimal landfill site. The suitable zone encompasses a small area (3%), predominantly located in the northeast region (Al-Mahalla), central region (Tanta), and northern region (Kotour). Conversely, the unsuitable area covers a substantial portion (72.7%) due to the agricultural nature of the governorate, high population density, and elevated groundwater levels. Furthermore, all existing landfill sites fall within unsuitable or low suitable areas, inflicting severe impacts on the nearby environment, public health, and groundwater integrity.

## Introduction

Municipal solid waste (MSW) refers to waste generated in cities that varies in terms of its characteristics and composition (Nanda & Berruti, 2021). The attributes and quantity of waste produced in a particular region are influenced by factors such as the lifestyles and living standards of the population and the growth rates of industrial and commercial activities, as well as the availability of natural resources in the area (Mohammed, 2019). Urban waste is typically categorized into organic and inorganic components (Nahman & Godfrey, 2010). The management of solid waste encompasses various processes, including waste incineration, landfill disposal, recycling, waste reduction, and reuse (Moeinaddini et al., 2010). Even when alternative waste management strategies are used, the establishment of landfill sites remains crucial for waste management. Particularly in countries that employ waste incineration, the presence of a suitable landfill site for residual waste is imperative (Alkaradaghi et al., 2019). Numerous factors, including public health concerns, heightened government regulations, environmental consciousness, municipal budgeting, growing political and social opposition to landfill site development, and limited availability of suitable land, all contribute significantly to the landfill siting process (Chabok et al., 2020). As a result, urban planners and authorities consider this process to be one of the most intricate challenges they encounter (Chabuk et al., 2017).

Many studies have investigated optimal landfill site selection utilizing multi-criteria decision-making (MCDM) methods in conjunction with geographic information systems (GIS). GIS integrates geographical data, such as maps, aerial imagery, and satellite images, with databases of qualitative, quantitative, and description information (Alkaradaghi et al., 2019), thereby rending GIS an effective tool for conducting site selection studies, particularly for landfills site assessment (Khorsandi et al., 2019). In other words, GIS is widely acknowledged as a highly capable and dependable technology for quickly analyzing and evaluating massive quantities of both geographic and non-spatial data (Kumar & Hassan, 2013).

Since the 1950s, MCDM approaches have become an essential tool, often used in tandem with GIS, to aid decision-makers (Sumathi, 2008). MCDM was developed to compute the weights of chosen factors (Özkan et al., 2019). Once appropriate weights are applied to the categories within each criterion map, an appropriate landfill site can be selected (Alkaradaghi et al., 2019; Randazzo et al., 2018). MCDM can be categorized into two groups, decision-making strategies in certainty and uncertainty environments, as shown in Table 1. The choice between these categories depends on the availability of crucial information. A decision is said to be made with certainty if the decision-maker possesses complete information about a complex situation (Malczewski, 1999). On the other hand, when the available information involves uncertainty, the decision is considered to be influenced by doubt (Malczewski, 1999). In the context of landfill site selection decisions, both in certain and uncertain environments, various strategies have been employed, as outlined in Table 1.

**Table 1:** The most popular MCDM methods

Multi-criteria decision-making (MCDM) methods	Decision-making techniques under certainty environment	Analytical hierarchy process (AHP)
		Analytic network process (ANP)
		The ratio scale weighting (RSW)
		Simple additive weighting (SAW)
		The straight rank sum method (SRS)
		Integrated AHP techniques
		Other integrated MCDM techniques
		Boolean logic (BMCDM)
	Decision-making techniques under uncertainty environment	Integrated fuzzy AHP techniques
		Integrated fuzzy ANP techniques
		Integrated fuzzy TOPSIS techniques
		Integrated fuzzy VIKOR techniques

Fuzzy multi-criteria decision-making (FMCDM) techniques have been integrated with GIS software to ascertain optimal sites for MSW landfills in Harlingen in South Texas (Chang et al., 2008a) and in the Lower Rio Grande Valley region of Texas (Chang et al., 2008b), USA. Gorsevski et al. (2012) used fuzzy membership functions, GIS software, AHP, and the ordered weighted average (OWA) methods to validate the optimal location for MSW in the Polog Region, Macedonia. The goal of this strategy was to highlight the various benefits of the OWA method’s weighting flexibility. This went beyond singling out a singularly effective method and extended to its capability to amalgamate disparate datasets and tailor specific criteria to different study areas. In Ariana Region (Tunisia), Aydi et al. (2013) used the theory of fuzzy sets, the weighted linear combination (WLC) technique, and AHP in a GIS environment to determine the most suitable landfill location, leading to the identification of five possible disposal sites.

Alkaradaghi et al. (2019) used GIS and ENVI software to select suitable landfill sites in the Sulaymaniyah Governorate, Iraq. Four methods of MCDM were utilized to calculate weights of thirteen criteria. Seven potential locations were chosen for landfill in the Sulaymaniyah Governorate using the most appropriate method. Randazzo et al. (2018) integrated GIS software with two methods of MCDM (AHP and SAW) to ascertain optimal MSW landfill sites in Sicily, Italy. The study underscored the effectiveness of multi-criteria decision analysis when coupled with GIS, presenting a robust methodology for identifying suitable sites for MSW. Delgado et al. (2008) compared three models of spatial decision support: Boolean logic (BMCDM), binary evidence, and an index of several category mappings. They showed that Boolean logic was less complex and more restrictive than the other two methods. In contrast, binary evidence and conflicting indexing techniques necessitated the assignment of weights to factors, mandating a suitability analysis. Barzehkar et al. (2019) used Boolean logic and fuzzy logic in order to choose landfill sites in the Sahar Khiz region of Iran. The study affirmed that fuzzy logic, especially when combined with the WLC approach, exhibited higher adaptability in handling conflicting human decisions. On the other hand, Boolean logic exhibited less accuracy than fuzzy logic in selecting ideal landfill sites for MSW in the case study. Numerous other studies utilized GIS and MCDM to choose landfill sites in different regions, such as the Pondicherry region in India (Sumathi, 2008), Shiraz city (Pasalari et al., 2019) and Salafchegan (Yousefi et al., 2018) in Iran, the West Mediterranean Region (Dereli & Tercan, 2021) and DOKAP Region (Yildirim et al., 2018) in Turkey, and Islamabad in Pakistan (Zarin et al., 2021). Several studies worldwide have employed the integration of GIS software and the AHP approach to guide the selection of optimal MSW landfill sites. Notable examples encompass a range of regions, such as Beijing in China (Wang et al., 2009), Serbia (Djokanović et al., 2016), Makkah in Saudi Arabia (Osra & Kajjumba, 2020), Gaza Strip (El Baba et al., 2015), Ahvaz in Iran (Chabok et al., 2020), and Iraq (Chabuk et al., 2016; Chabuk et al., 2019). These studies used different buffer zones for different criteria due to environmental circumstances and the characteristics of the field of research, as shown in Table 2. Furthermore, a summary of recent global studies on landfill site selection is provided in Table 3.

**Table 2:** Suggestion of buffering zones for sub-criteria in the literature

Criteria	References	Description
Distance to surface water	Delgado et al. (2008); Feyzi et al. (2019); Alkaradaghi et al. (2019)	Minimum 1 km away from surface water resources
	Sadek et al. (2006)	Minimum 1 km away from lakes, 0.5 km away from rivers, 0.25 km away from streams, 0.15 km away from other running water
	Gorsevski et al. (2012)	Minimum 0.5 km away from lakes and potable water sources, 0.3 km away from rivers
	Bosompem et al. (2016)	Minimum 0.5 km and maximum 2.5 km away from surface water
	Saatsaz et al. (2018)	Minimum 0.25 km away from surface water
Distance to road	Wang et al. (2009)	Minimum 0.5 km away from main roads
	Gorsevski et al. (2012)	Minimum 2 km away from main roads
	Alkaradaghi et al. (2019)	Minimum 0.5 km and maximum 5 km away from main roads
	Bosompem et al. (2016)	Minimum 0.5 km and maximum 4 km away from main roads
	Feyzi et al. (2019)	Minimum 0.3 km away from roads
Distance to urban areas	Bosompem et al. (2016); Osra and Kajjumba (2020)	Minimum 5 km away from urban centers
	Chabuk et al. (2019); Alkaradaghi et al. (2019)	Minimum 5 km and maximum 15 km away from urban centers
	Feyzi et al. (2019)	Minimum 1 km away from city centers
Slope	Demesouka et al. (2013)	Less than 10%
	Gorsevski et al. (2012)	Less than 30%
	Alavi et al. (2013); Feyzi et al. (2019)	Less than 45%
Land use, land cover	Demesouka et al. (2013)	Forested lands are not suitable
	Chabuk et al. (2017)	Unused land is the most suitable
	Delgado et al. (2008)	Forested lands are not suitable
Distance to railways	Chabuk et al. (2017); Chabuk et al. (2019)	Minimum 500 m away from railways
Distance to power lines	Alkaradaghi et al. (2019)	Minimum 30 m away from power lines
	Yousefi et al. (2018)	Minimum 200 m away from power lines
Distance to villages	Bosompem et al. (2016)	Minimum 0.5 km and maximum 2 km away from villages
	Alkaradaghi et al. (2019); Chabuk et al. (2019)	Minimum 1 km away from villages
Soil type	Alanbari et al. (2014); Chabuk et al. (2019)	Silt clay is highly suitable for landfill
Elevation	Chabuk et al. (2019)	Between 11 and 34 m (a.m.s.l.)
	Ali et al. (2021)	Between 10 and 28 m (a.m.s.l.)
Depth to groundwater	Delgado et al. (2008); Chabuk et al. (2017)	Minimum 10 m
	Effat and Hegazy (2012)	Minimum 5 m
	Chabuk et al. (2017)	Minimum 1.5 m
	Chabuk et al. (2019)	The greatest value of groundwater depth received the highest possible rating, and the smallest value (the shallowest) received the lowest possible rating

**Table 3:** Summary of recent studies on landfill site selection in the world

Study	Region	Criteria	MCDM	Main findings
Chang et al. (2008)	Harlingen in South Texas, USA	RI, LA, WE, LU, RO, GWW, UA, SO, ELE, CCD	FMCDM	Seven landfill sites were determined
Delgado et al. (2008)	Cuitzeo Lake Basin, Mexico	LA, UA, AP, LU, CI, PS, AGFF, SO, SL, RO	BMCDM, binary evidence, and overlapping index	The Boolean logic approach is the best option out of the three
Sumathi (2008)	Pondicherry region, India	SW, RO, LU, UA, PW, PSS, GW, ELE, SO, DF, NGWQ, AQI	AHP	17 landfill sites were determined
Wang et al. (2009)	Beijing, China	SW, GW, AP, LU, SO, SL, RO, PL, FL	AHP	Optimal landfill sites were determined
Gorsevski et al. (2012)	Polog Region, Macedonia	SL, ELE, RI, LA, DS, LU, AGFF, UA, RO, PSS, DF	FMCDM and OWA	Landfill sites were determined
Shahabi et al. (2014)	Saqqeq city, Kurdistan	SL, ER, AGFF, GW, SW, GWW, AP, UA, PSS, LU, RO, PL	BMCDM and AHP	AHP has the power to make better decisions for locating landfills than Boolean logic
Djokanović et al. (2016)	Serbia	SL, GW, SO, SW, RI, LU, PL, PSS, ELE, AP, DF	AHP	62.3% of the sites are unsuitable, 13.5% are poorly suitable, 12.1% are moderately suitable, and 12.1% are the most suitable
Randazzo et al. (2018)	Sicily, Italy	SL, LU, RO, SW, UA, AGFF, AAR, SO	AHP and SAW	There have been identified a number of appropriate locations for MSW landfills
Zarin et al. (2021)	Islamabad, Pakistan	RI, RA, SW, RI, SO, SL, UA, LU, SE, PD	AHP and FMCDM	AHP and FMCDM identified an area of 47 km^2^ and 36 km^2^ as high suitable area, respectively
Ali et al. (2021)	Bardhamman, India	RI, GW, GWW, DP, ELE, LU, UA, PSS, RO, PL, PD	AHP, FMCDM, and FTOPSIS	Two sites were selected as the most suitable for proposing new landfill sites

Durlević et al. (2023) employed a combination of GIS and fuzzy MCDM techniques to determine the optimal landfill site for MSW in the city of Kraljevo in Serbia. The study encompassed the collection of 15 environmental factors, the selection of ten diverse locations for landfill, and the ranking assignment for the final chosen sites through fuzzy analysis. The findings of this research indicated that the A4 site, a sanitary landfill location, emerged as the most suitable option, offering a spacious area of about 569 ha and a favorable proximity to the urban area at roughly 8 km. This study pioneered the integration between GIS and fuzzy AHP, alongside multi-objective analysis techniques to find the most appropriate site for landfill. The outcomes of this study hold the potential for enhancing safe waste disposal management practices in Kraljevo, Serbia. In a similar vein, Abdo et al. (2023) integrated GIS with MCDM techniques for the selection of optimal MSW landfill site in the Safita area (Syria). Physical, economic, and technological factors were considered to face the challenges posed by the region’s growing population, development, and urbanization. By employing 13 criteria informed by prior studies, the researchers generated a suitability landfill map. The results of this research offer valuable guidance for identifying new landfill locations. The methodologies employed in this research demonstrate potential adaptability for integrated waste management solutions in other regions of Syria.

In Egypt, few studies have investigated landfill sites for MSW in different areas using the combination of GIS and AHP, as shown in Table 4. For example, El Alfy et al. (2010) identified seven key parameters for landfill site selection in Mansoura, namely surface water, residential areas, railway proximity, archaeological significance, sensitive areas, road accessibility, and urban zones. The findings indicated that approximately 2.9% of the total area exhibited potential for suitable land for landfill purposes. Effat and Hegazy (2012) determined optimal landfill sites for MSW in Sinai by producing a suitability index map for three categories: environmental, social, and economic. Abd-El (2015) discovered appropriate spots to establish new landfills in rapidly developing tourist destinations along the Red Sea’s coastal desert districts.

**Table 4:** Summary of recent studies on landfill site selection in MENA

Study	Region	No. of criteria	MCDM	Main findings
El Alfy et al. (2010)	Mansoura city, Egypt	SW, HP, UA, RA, RO, MA, AP	AHP	Only 2.9% of the total area can be considered suitable land
Effat et al. (2012)	Sinai region, Egypt	SO, GW, WE, AGFF, CZ, UA, SL, PL, RO, AP, PSS	BMCDM and AHP	Optimal locations were determined for MSW landfill
Aydi et al. (2013)	Ariana Region, Tunisia	SO, ELE, SL, CZ, DTIL, GW, SW, WE, RI, CA, LU, RO, UA	FMCDM	Five landfill sites were determined
El Baba et al. (2015)	Gaza, Palestine	LU, SO, GW, RO, ELE, AAR	AHP	For future landfills, approximately 5.48% of the total area is acceptable
Abd-El (2015)	Red Sea, Egypt	UA, AGFF, RO, AP, CZ, WE, GW, SL, ELE,	AHP	Three suitable landfill sites were determined
Bahrani et al. (2016)	Shabestar, Iran	SL, SO, ELE, AGFF, GW, UA, SW, LU, HP, GE	FMCDM and AHP	A 6.2% portion of the research area is well suited for MSW
Chabuk et al. (2017)	Al-Musayiab Qadhaa, Babylon, Iraq	GW, RI, ELE, SO, SL, RO, UA, HP, PL, LU, ALU, RA, VI, GP	AHP and SAW	Optimal locations were determined for MSW landfill
Alkaradaghi et al. (2019)	Sulaymaniyah Governorate, Iraq	SO, LU, ELE, SL, RI, GW, AGFF, RO, UA, VI, HP, GP, PL,	AHP, SAW, SRS, and RSW	Seven locations were selected for landfill within the most suitable type
Barzehkar et al. (2019)	SaharKhiz Region, Iran	FL, SL, SO, AGFF, RI, LA, CZ, PSS, WE, UA, RO, PL, GP, GW	FMCDM, BMCDM, and AHP	Fuzzy logic has more accuracy than Boolean logic
Chabok et al. (2020)	Ahvaz, Iran	GW, RI, ELE, SO, SL, RO, UA, HP, PL, LU, ALU, RA, VI, GP	FMCDM	Eleven areas were chosen as feasible MSW landfill sites
Osra et al. (2020)	Makkah, Saudi Arabia	HP, UA, GW, PSS, SL, WD, RO, AP, AAR, RP, LU	AHP	Six sites were determined for landfill from 2020 to 2030

*RI* rivers, *CA* canals, *LA* lakes, *WE* wetland, *LU* land use, *RO* roads, *GW* groundwater, *GWW* groundwater wells, *UA* urban areas, *SO* soil, *ELE* elevation, *CCD* county census data, *AP* airports, *CI* communication infrastructure, *PS* petrochemical storage plant, *AGFF* active geologic fractures and faults, *SL* slope, SW surface water, *PW* proximity to wasteland, *PSS* proximity to sensitive sites, *DF* degree of infiltration, *GWQ* groundwater quality, *AQI* air quality index, *PL* price of land, *FL* forest land, *DS* distance of springs, *ER* erosion, *PL* power lines, *AAR* average annual rainfall, *CZ* coastal zone, *DTIL* depth to impermeable layer, *HP* historical places, *RA* railway, *MA* military areas, *WD* wind direction, *ALU* agricultural land use, *VI* villages, *GP* gas pipelines, *G* geology, *RP* rock profile, *FL* flooding, *SE* settlements, *DP* distance from pipeline, *PD* population density

Al-Gharbia Governorate, situated in the central part of the Nile Delta in Egypt, is grappling with the adverse consequences of haphazardly chosen solid waste disposal sites, which leads to severe environmental problems. Presently, only eight landfills for MSW are scattered throughout the cities of Al-Gharbia. Regrettably, these sites fall short of meeting fundamental scientific and environmental standards. To the best of the authors’ knowledge, none of the previous publications have addressed the evaluation of the existing landfill sites in the governorate. Therefore, this study aims to fulfill two primary objectives: firstly, identifying optimal landfill locations within Al-Gharbia Governorate, and secondly, validating the suitability of the existing landfill sites. The study’s framework involves the selection of eleven distinct criteria encompassing both natural and artificial aspects. These criteria include groundwater levels, rivers, canals, soil types, urban centers, villages, land use patterns, elevation variations, slope gradients, road networks, power lines, and railways. To achieve these goals, the study adopts an integrated approach, utilizing ArcGIS in conjunction with four MCDM methods: AHP, RSW, SRS, and Boolean logic methods. These methodologies collectively contribute to the generation of four distinct suitability landfill site index maps. Ultimately, the synthesis of three methods AHP, RSW, and SRS will culminate in the derivation of the final suitability index map, offering a comprehensive assessment of optimal landfill locations in Al-Gharbia Governorate.

## Study area

**Fig. 1:**
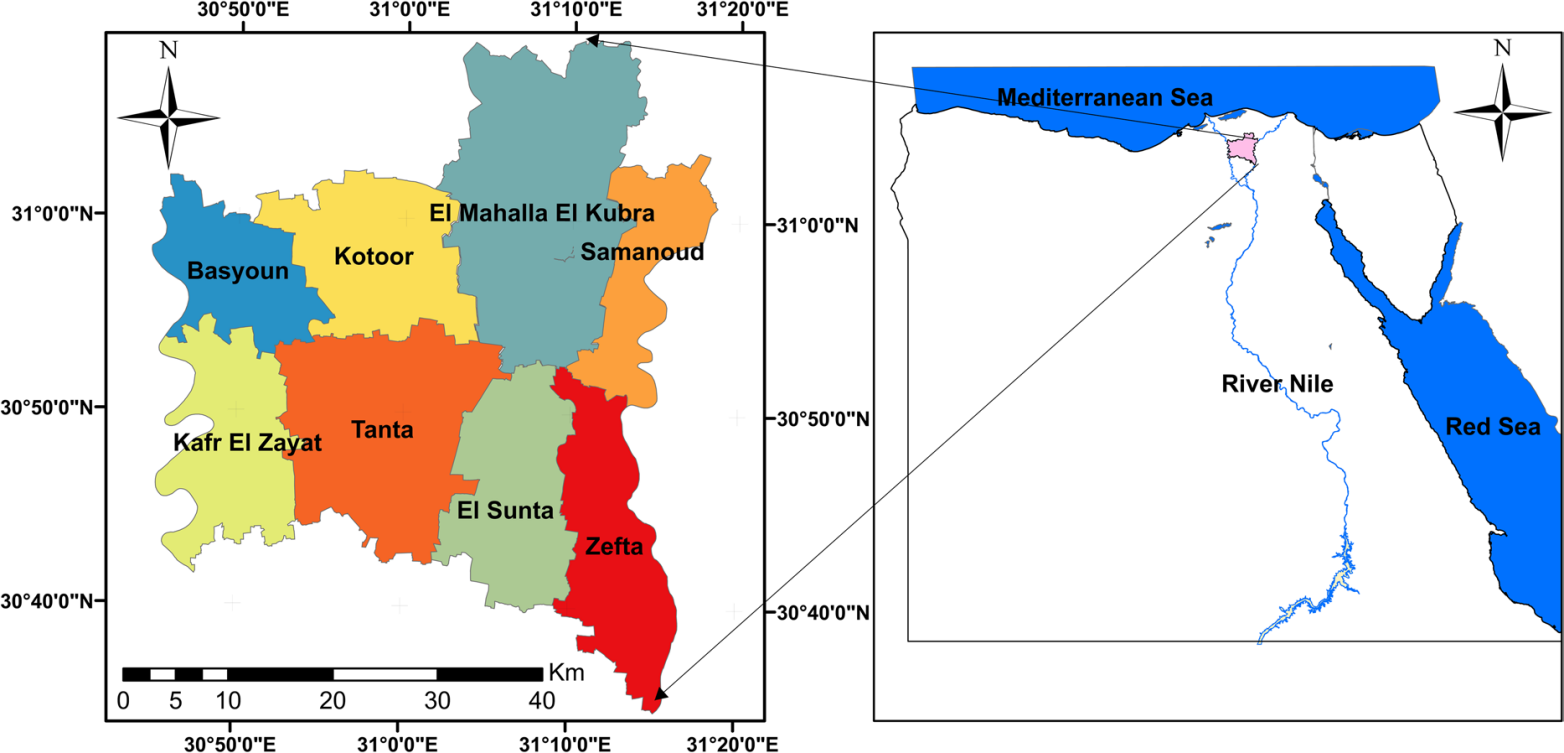
The map of Al-Gharbia Governorate

Al-Gharbia Governorate is strategically situated in the middle of the Nile Delta, spanning latitudes 30° 35′, 31° 10′ N and longitudes 30° 45′, 31° 15′ E, as shown in Fig. 1. Its total area covers 1942 km^2^, with varying land uses including cultivated area (1658 km^2^), residential area (214 km^2^), and vacant land area (70 km^2^) (Khalifa et al., 2019). The land of the governorate is sedimentary in nature, formed over millennia by the deposition of silt in the Nile Delta, and its soil classification ranges from clay to muddy clay (Garzanti et al., 2018). Al-Gharbia Governorate is mainly agricultural, owing to the quality and fertility of its agricultural lands. It encompasses 8317 main villages and 1249 minor hamlets (Khalifa et al., 2019). Therefore, Al-Gharbia Governorate is one of the most important rural Governorates in Egypt, with an agricultural area of about 394 thousand acres, constituting 86% of the Al-Gharbia’s overall area. Approximately 70% of the population resides in rural areas and is largely engaged in agriculture (Willcocks & Brown, 1899). It ranks as the third most populous governorate in Egypt, with a population density of 2608 persons/km^2^. As of December 7, 2021, the governorate’s population stands at 5,339,737 according to the CAPMAS (http://www.capmas.gov.eg), making a doubling since 1976. In winter, the average temperature drops to 15.2 °C, while in summer, it rises to 27.9 °C (Gado et al., 2021). The annual rainfall ranges between 50 and 79 mm (Gado et al., 2021; Gado and El-Agha, 2021). The rate of groundwater recharge from rainfall ranged from 0.0 to 20 mm/winter season, in the central part of the Nile Delta (Armanuos et al., 2016; Armanuos et al., 2017).

## Material and methods

**Fig. 2:**
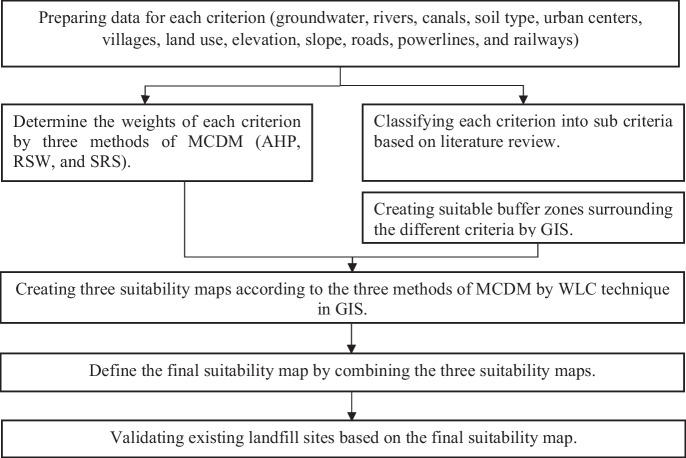
A diagram outlining the procedure followed to determine the optimal landfill sites

To select an appropriate location for landfill, GIS software will be utilized to create different layers representing various criteria (Table 5) for the research area. The research methodology can be summarized as follows (Fig. 2):

1. Criterion selection and data preparation: gathering data from available sources for each criterion
2. Criterion classification: categorizing each criterion into sub-criteria based on a comprehensive literature review
3. Buffer zone establishment: defining suitable buffer zones around different criteria using GIS
4. Criterion weight determination: assigning weights to each criterion using three methods of MCDM (AHP, RSW, and SRS)
5. Suitability maps creation: generating three suitability maps using the WLC technique in GIS, corresponding to the three MCDM methods
6. Final suitability map: integrating the three suitability maps to produce a comprehensive map indicating overall appropriateness
7. Validation of existing landfill sites: using the final suitability map to assess and validate the suitability of existing landfill sites

**Table 5:** Data collection sources

No	Criteria	Source
1	Rivers	USGS Earth Explorer (https://earthexplorer.usgs.gov)
2	Roads	
3	Elevation	
4	Urban area	Google Earth® (https://www.google.com.eg/intl/ar/earth/)
5	Soil type	Khalifa et al. (2019)
6	Slope	Utilizing GIS, DEM was transformed into a slope map
7	Power line	Open Street Map in GIS® (https://www.esri.com/en-us/what-is-gis/overview)
8	Villages	Google Earth® (https://www.google.com.eg/intl/ar/earth/)
9	Land use	USGS Earth Explorer, Landsat 8 (OLI)
10	Groundwater	Ministry of Water Resources and Irrigation (personal communication)
11	Canals	Google Earth® (https://www.google.com.eg/intl/ar/earth/)
12	Existing landfill sites	Ministry of Environment (personal communication)

### Selecting criteria and preparing data for each criteria

**Fig. 3:**
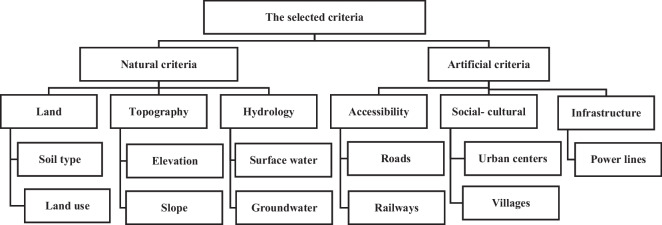
Illustration of the different criteria used to determine the optimal location for landfill

**Fig. 4:**
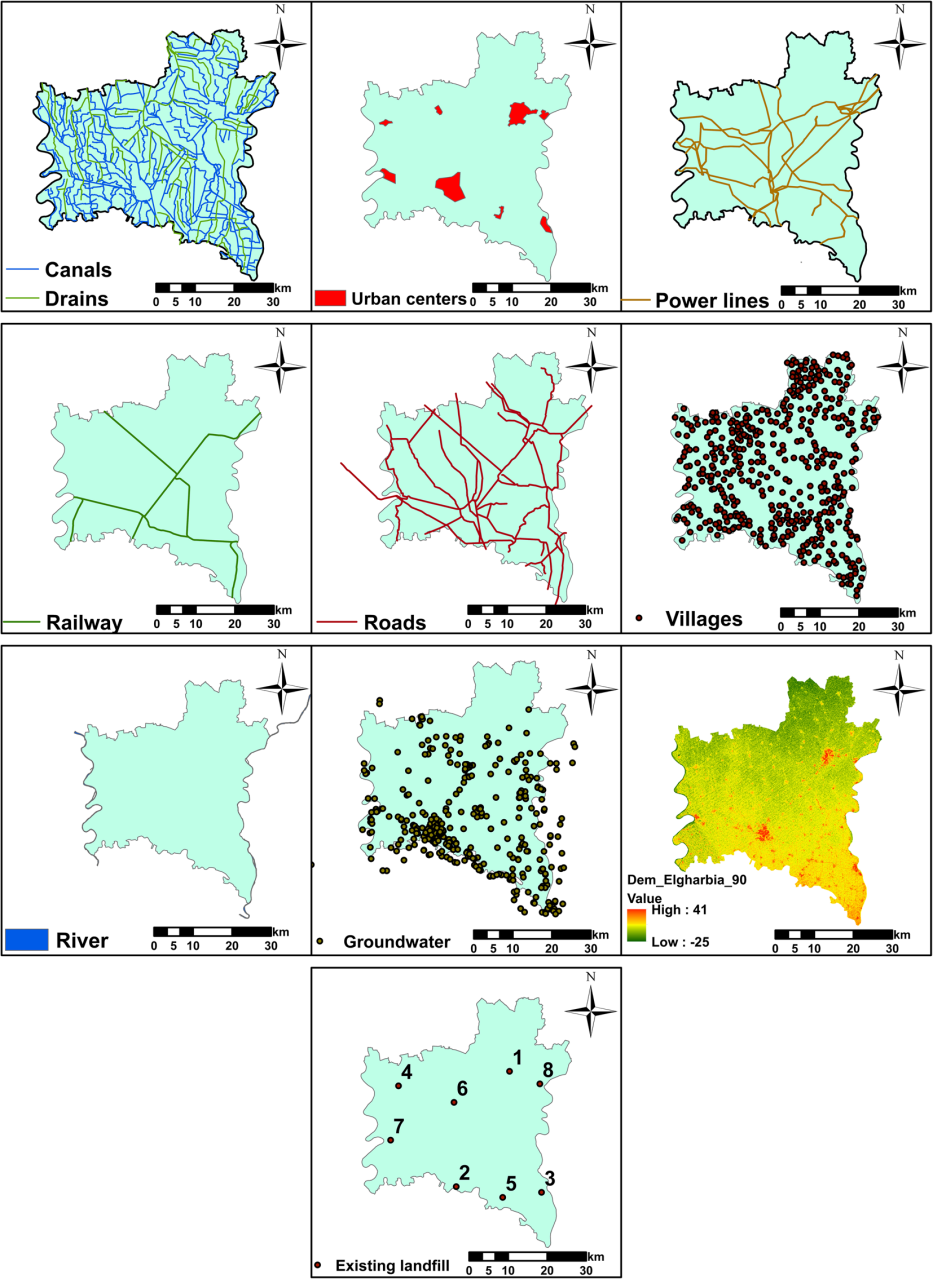
Criteria maps used to determine the optimal landfill locations

As a general rule, the selection of criteria is based on expert recommendations in the field, previous research, available data, and the unique characteristics of the study region. In this study, data for different criteria were collected from global organizations and government entities (Table 5). These criteria can be broadly categorized into natural and artificial factors, as shown in Fig. 3. Groundwater levels and existing landfill sites were procured from government sources. Surface water, land use information, and road networks were obtained from the USGS Earth Explorer platform. An elevation map was derived from digital elevation model (DEM) data, which was subsequently transformed into a slope map. Urban areas, canals, and villages were delineated using Google Earth imagery, as shown in Fig. 4.

### Buffer zones and rating of sub-criteria

Each criterion establishes buffer zones encompassing significant locations or specific geographic characteristics to protect them from the potential impact of landfill sites. To calculate the minimum and maximum distances from landfill sites, each criterion is defined with a designated distance, considering factors such as environmental considerations, cost implications, public health concerns, and compliance with governmental regulations (Alkaradaghi et al., 2019; Chabok et al., 2020). Following the subdivision of each criterion into sub-criteria, all criteria were allotted a rating score spanning from 0 to 10. This assignment was guided by considerations encompassing pertinent laws, regulations, restrictions, and stipulated requirements, along with insights from literature studies and expertise from scientific professionals (Alkaradaghi et al., 2019) (Table 6).

**Table 6:** Ratings of the sub-criteria and descriptions of the buffer zones

No.	Criterion	Buffer zone	Sub-criteria ratings
1	Depth to groundwater level (m)	2–5	0
		5–8	4
		8–10	6
		>10	10
2	Distance to surface water (rivers, canals, and drains) (m)	0–1000	0
		>1000	10
3	Elevation (m) (a.m.s.l.)	0–5	0
		5–7	3
		7–10	8
		>10	10
4	Slope (degree)	0–10	10
5	Soil types	Silt clay	7
6	Land use	Urban areas	0
		Rivers	0
		Agricultural land	0
		Unused area	10
7	Distance to roads (km)	0–500	0
		500–1000	7
		1000–2000	10
		2000–3000	5
		>3000	3
8	Distance to railways (m)	0–500	0
		>500	10
9	Distance to urban areas (km)	0–5	0
		5–10	10
		10–15	7
		>15	4
10	Distance to villages (km)	0–1	0
		>1	10
11	Distance to the power lines (m)	0–300	0

#### Depth to groundwater

**Fig. 5:**
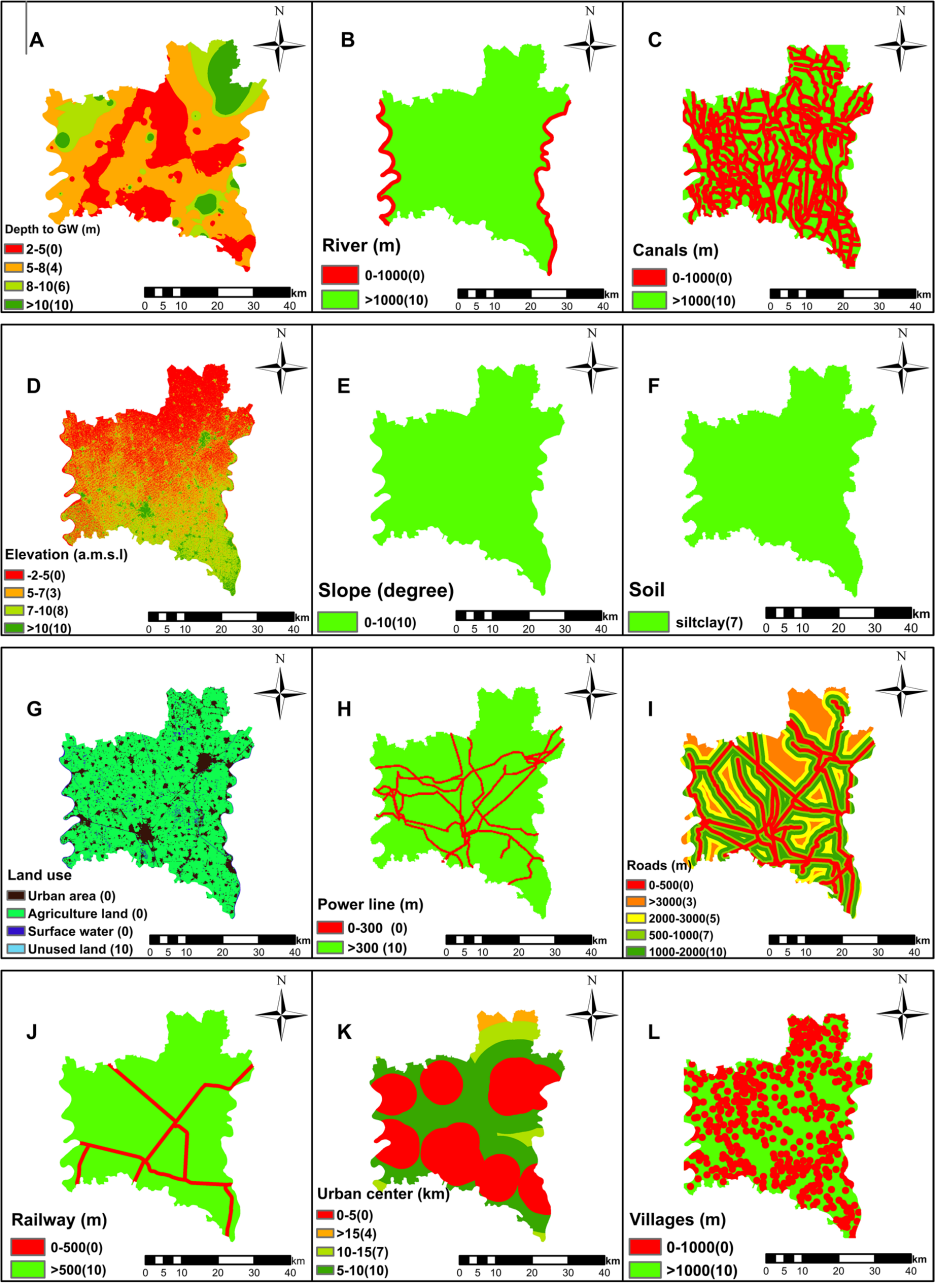
Classified maps of Al-Gharbia Governorate for **A** depth to the groundwater, **B** distance to the rivers, **C** distance to the canals and drains, **D** elevation, **E** slope, **F** soil types, **G** land cover, **H** distance to the power lines, **I** distance to the roads, **J** distance to the railway, **K** distance to the urban areas, and **L** distance to the villages

A prominent environmental concern associated with landfill sites is the potential contamination of groundwater due to the migration of leachate and pollutants originating from landfill (Alkaradaghi et al., 2019). To mitigate the risk of leaching from solid waste and safeguard aquifers from pollution, landfills should be situated in an area with a significant depth of groundwater (Chabuk et al., 2019). In most parts of Al-Gharbia Governorate, the depth to groundwater varies between 2 and 10 m. The groundwater levels of Al-Gharbia Governorate were collected from the groundwater sector of the Ministry of Water Resources and Irrigation (MWRI) in Egypt (Table 5). To produce a comprehensive groundwater level map for Al-Gharbia Governorate, 373 wells were integrated into the GIS framework using interpolation tools to generate the raster map. In alignment with the study’s parameters outlined in Table 2, a buffer zone of 5 m was established for this criterion (Fig. 5A and Table 6).

#### Distance to surface water

To prevent the contamination of surface water, a buffer zone with a width of 1000 m was established between the landfill and the surface water (Alkaradaghi et al., 2019; Feyzi et al., 2019). Each region in the vicinity of less than 1000 m was assigned a value score of 0, while all regions with distances larger than 1000 m were assigned a value score of 10 (Fig. 5B, C; and Table 6).

#### Elevation

Elevation plays a key role in the site selection for a landfill. It should be high enough to mitigate the risk of waste leachate seepage and flooding. However, it should also strike a balance to avoid excessive costs related to waste transportation and inadequate protection against the prevailing winds (Alkaradaghi et al., 2019). In this study, elevations below 5 m above the Mediterranean mean sea level (a.m.s.l.) were assigned a score of 0. Elevations falling within the ranges of 5 to 7 m, 7 to 10 m, and above 10 m were assigned respective values of 3, 8, and 10, as displayed in Fig. 5D and Table 6.

#### Slope

Many natural phenomena are influenced by the land slope, including soil moisture, soil erosion, and the rate of both surface and underground flow (Osra & Kajjumba, 2020). Therefore, the land slope is essential for the development and process of landfills (Gorsevski et al., 2012). Wang et al. (2009) considered locations with a slope of 0–10% to be most suitable, while a slope of 40–50% is not appropriate for landfills. In this research, the whole region was assigned a score of 10, where the slope of the study region is not larger than 10%, as shown in Fig. 5E and Table 6.

#### Soil type

A crucial element in landfill site selection is the rate of infiltration, as it directly influences the potential risks of groundwater pollution (Alkaradaghi et al., 2019; Sumathi, 2008). Soils characterized by high values of permeability (e.g., sandy and sandy loam) are deemed inappropriate for landfill sites, while soils with low to medium permeability (e.g., sandy clay) are ideal for this purpose (Chabok et al., 2020). In this study, there is a single soil type (i.e., silty clay) in Al-Gharbia Governorate (Khalifa et al., 2019) and it has been attributed a rating of 7 (Khorsandi et al., 2019), as shown in Fig. 5F and Table 6.

#### Land use

In general, areas characterized by lower public significance encounter comparatively less opposition to landfill construction, often receiving greater acceptance than areas of higher public value (Gorsevski et al., 2012). The classification of land uses was created through the utilization of remote sensing technologies and Landsat images (ENVI software). In this study, land use was categorized into four groups (water bodies, building, agricultural land, and unused land) (Chabuk et al., 2019), as shown in Fig. 5G and Table 6.

#### Distance to power lines

Landfill sites should be situated at a sufficient distance from power lines to shield them from potential damage (Moeinaddini et al., 2010). In the current study, a buffer zone, spanning 300 m in width (Yousefi et al., 2018), was established between the landfill area and the power lines, as shown in Fig. 5H and Table 6.

#### Distance to roads

There should be an appropriate distance between roadways and landfills to ensure the safety of motorists, protecting them from accidents caused by road debris during high winds (Baban & Flannagan, 1998; Demesouka et al., 2013), and also to prevent any adverse visual effects (Chabok et al., 2020). However, landfill locations should not be overly distant from roads due to economic considerations. In this research, a rating system was applied to different buffer zones. The 500-m buffer zone was given a rating of 0, the buffer zone between 500 and 1000 m received a rating of 7, and the buffer zone between 1000 and 2000 m received a rating of 10. On the other hand, the buffer zones between 2000 and 3000 m and beyond 3000 m were assigned ratings of 5 and 3, respectively (Alkaradaghi et al., 2019) (Fig. 5I and Table 6).

#### Distance to railways

The distance between railways and landfill locations should be considerable to minimize the risk of land subsidence and aesthetic effects (Alkaradaghi et al., 2019; Feyzi et al., 2019). In this research, a buffer zone of 500 m on either side of the railway was given a rating of 0, while a score of 10 was assigned in other cases (Djokanović et al., 2016), as shown in Fig. 5J and Table 6.

#### Distance to urban areas

The distance between urban areas and landfill locations should be substantial enough to ensure the protection of people and the surrounding environment from the negative impacts of landfills, such as diseases, insects, and odors (Chabuk et al., 2019). However, it is important to strike a balance and avoid placing landfills excessively far from urban areas due to economic considerations, including transportation costs (Abd-El, 2015). In the current research, a buffer zone of 5 km wide was established between the landfill and the adjacent urban areas (Alkaradaghi et al., 2019; Effat & Hegazy, 2012). The buffer zone spanning from 5 to 10 km received a rating of 10, the buffer zone between 10 and 15 km received a rating of 7, and the buffer zone exceeding 15 km received a rating of 4, as shown in Fig. 5K and Table 6.

#### Distance to villages

To protect the residents of the numerous communities scattered throughout Al-Gharbia Governorate from the effects of landfill locations, buffer zones of 1 km wide have been allocated around each village, marked with a score of zero (Table 2). Alternatively, a rate of 10 has been assigned to buffer zones exceeding 1 km in width (Fig. 5L and Table 6).

### Multi-criteria decision-making (MCDM) methods

MCDM methods play a crucial role in structuring and resolving planning and decision-making challenges characterized by multiple influencing factors. They offer valuable assistance to decision-makers when grappling with such complexities. Often, such challenges lack a solitary optimal solution, underscoring the importance of incorporating the viewpoints of decision-makers to differentiate among potential options (Majumder, 2015). In this study, four techniques of MCDM were used: the analytical hierarchy procedure (AHP), RSW, SRS, and Boolean logic.

#### Analytical hierarchy procedure (AHP)

The analytical hierarchy process (AHP), introduced by Saaty (1980), stands as one of the most extensively used methodologies within multiple criteria decision-making (MCDM). Its purpose is to aid decision-making through the amalgamation of available data and expert opinions (Tercan et al., 2022). Recognizing that human judgment can occasionally display inconsistencies, the AHP approach mitigates such inaccuracies by utilizing pairwise comparisons instead of direct assessments of scores and weights for factors (Nasiri et al., 2022; Romero-Ramos et al., 2023). The relative significance among different criteria was determined by employing a 9-point numerical scale, based on literature studies, as displayed in Table 7. To calculate the relative weights of criteria in the AHP approach, a pairwise matrix of correlations was generated using Eq. 1 (Table 8). For all criteria, the eigenvector (*Eg*_i_) for each row in the matrix was calculated using Eq. 2, and subsequently, the priority vectors (*Pr*_i_) were calculated using Eq. 3 (Barzehkar et al., 2019). The evaluation of the acceptability of criteria comparisons within the pairwise matrix involves calculating the consistency ratio (CR), following these steps:

1. The consistency index (CI) is calculated utilizing Eq. 6 involving the calculation of *λ* and *λ*_total_ via Eqs. 4 and 5, respectively (Chabuk et al., 2017). In this study, the values of CI and *λ*_total_ are 0.148 and 12.48, respectively.
2. The random inconsistency index (RI) is determined using values from Table 9, which are dependent on the matrix size (*n*). In this case, for eleven criteria, the RI value is 1.51.
3. The CR is determined by dividing the calculated CI by the determined RI, as shown in Eq. 7. A CR value equal to or lower than 0.1 is considered acceptable for consistency. In this research, the CR value is 0.098.

**Table 7:** Relative relevance grading scale for pairwise comparison (Alkaradaghi et al., 2019)

Numerical value	Definition
1	Equal significance
2	Of approximately equal to moderate significance
3	Medium significance
4	Medium to significant importance
5	Strong significance
6	Highly important to very highly important
7	Very extremely significant
8	Very to extremely strong significant
9	Extreme significant

**Table 8:**
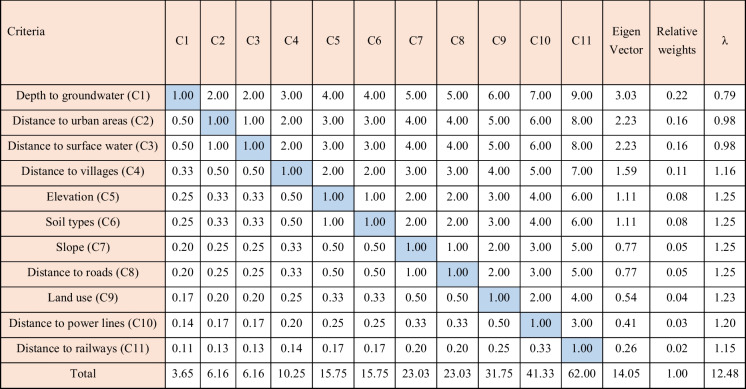
Pairwise comparison matrix for landfill siting using AHP

**Table 9:** Random inconsistency index for different values of *n* (Alkaradaghi et al., 2019)

N	1	2	3	4	5	6	7	8	9	10	11	12	13
RI	0.0	0.0	0.58	0.9	1.12	1.24	1.32	1.41	1.45	1.49	1.51	1.48	1.56

1 $${a}_{\mathrm{ij}}=\frac{1}{a_{\mathrm{ji}}}$$

2 $${\mathrm{Eg}}_{\mathrm{i}}={\left({a}_{11}\times {a}_{12}\times {a}_{13}\times {a}_{14}\dots \dots ..\times {a}_{1\mathrm{n}}\right)}^{\frac{1}{\mathrm{n}}}$$

3 $${\Pr}_{\mathrm{i}}=\frac{{\mathrm{Eg}}_{\mathrm{i}}}{\sum_{\mathrm{i}=1}^{\mathrm{n}}{\mathrm{Eg}}_{\mathrm{i}}}$$

4 $$\lambda ={\Pr}_{\mathrm{i}}\sum_{\mathrm{i}=1}^{\mathrm{n}}{\mathrm{a}}_{\mathrm{i}\mathrm{j}}$$

5 $${\lambda}_{\mathrm{total}}=\sum_{\boldsymbol{i}=\textbf{1}}^{\boldsymbol{n}}\lambda$$

6 $$\mathrm{CI}=\frac{\left({\lambda}_{\mathrm{total}}-\mathrm{n}\right)}{\left(\mathrm{n}-1\right)}$$

7 $$\mathrm{CR}=\frac{\mathrm{CI}}{\mathrm{RI}}$$

where *a*_ij_ is a component of the pairwise comparison matrix for row *i* and column *j*, Eg_i_ is the eigenvalue for the row *i*, *n* is the matrix size, and Pr_i_ is the priority vector for row *i*.

#### Ratio scale weighting (RSW) method

In the RSW method, relative weights were calculated according to the importance of each factor relative to the others. The significance among criteria was determined based on published research (Alkaradaghi et al., 2019; Chabuk et al., 2017). A value of 100 was assigned to the most crucial criterion as the baseline for the weights of the other criteria. Values less than 100 were assigned proportionally to criteria with lower significance, reflecting their relative importance compared to others (Alkaradaghi et al., 2019; Chabuk et al., 2017). Then, the scale value of each criterion was divided by the value of the least significant criterion to calculate the ratio weights (*R*_i_) (Chabuk et al., 2017). Finally, the assigned relative weights (*W*_i_) for the used criteria were determined using Eq. 7, as shown in Table 10.

8 $${\boldsymbol{W}}_{\textbf{i}}=\frac{{\boldsymbol{R}}_{\textbf{i}}}{\sum_{\boldsymbol{i}=\textbf{1}}^{\boldsymbol{n}}{\boldsymbol{R}}_{\textbf{i}}}\kern0.75em \boldsymbol{i}=\textbf{1},\textbf{2}\dots .,\boldsymbol{n}$$

where *n* is the number of criteria.

**Table 10:** The relative weights of criteria utilizing the RSW technique

Criteria	Scale value	Ratio weights (*R*_i_)	Relative weights (*W*_i_)
Depth to groundwater	100	20	0.225
Distance to urban areas	74	14.8	0.166
Distance to surface water	73	14.6	0.164
Distance to villages	52	10.4	0.117
Elevation	35	7	0.079
Soil types	35	7	0.079
Slope	23	4.6	0.052
Distance to roads	23	4.6	0.052
Land use	15	3	0.034
Distance to power lines	10	2	0.022
Distance to railways	5	1	0.011
Total	Min = 5	89	1.000

#### Straight rank sum (SRS) method

The SRS method is a straightforward technique employed to compute criteria weights by arranging criteria in a decreasing order of relative significance ranging from the most to the least important, based on earlier studies. Subsequently, the relative weights (*W*_i_) of the criteria (Table 11) are normalized using Eq. 9 (Alkaradaghi et al., 2019; Effat & Hegazy, 2012).

9 $${W}_{\mathrm{i}}=\frac{\left(n-{r}_{\mathrm{i}}+1\right)}{\sum_{\boldsymbol{i}=\textbf{1}}^{\boldsymbol{n}}\left(\boldsymbol{n}-{r}_{\mathrm{i}}+\textbf{1}\right)}$$

where *r*_i_ is the ranking position for each criterion.

**Table 11:** The relative weights of criteria utilizing SRS approach

Criteria	Ranking	*n* − *r*_i_ + 1	Relative weights
Depth to groundwater	1	11	0.167
Distance to urban areas	2	10	0.152
Distance to surface water	3	9	0.136
Distance to villages	4	8	0.121
Elevation	5	7	0.106
Soil types	6	6	0.091
Slope	7	5	0.076
Distance to roads	8	4	0.061
Land use	9	3	0.045
Distance to power lines	10	2	0.030
Distance to railways	11	1	0.015
Total	66	66	1

#### Boolean method

**Fig. 6:**
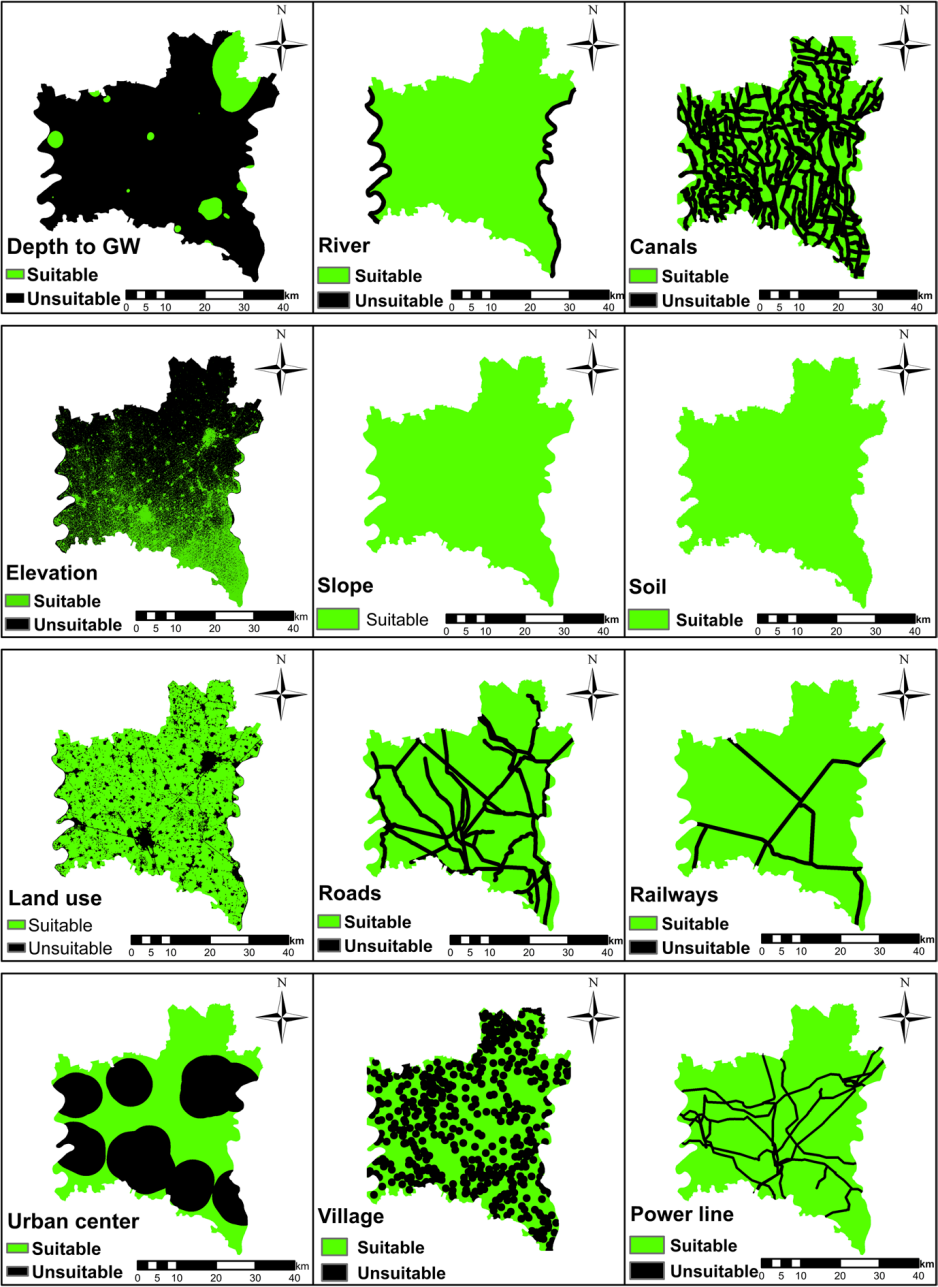
Suitability maps of Al-Gharbia Governorate derived by Boolean method using Table 12

**Fig. 7:**
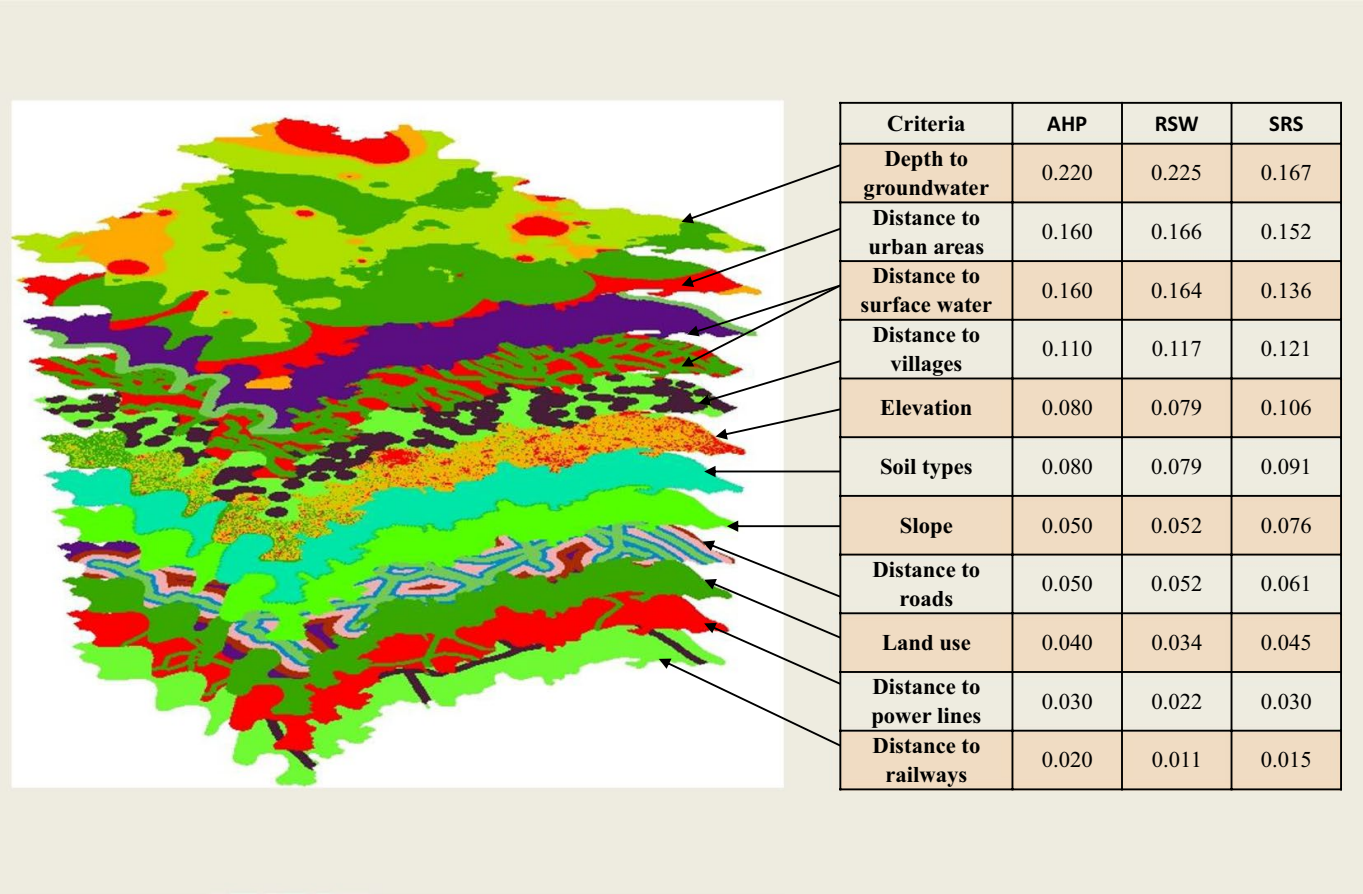
The integration of the relative weights of the criteria and the rating values by the WLC method along with the relative weights of the criteria of the three methods

**Fig. 8:**
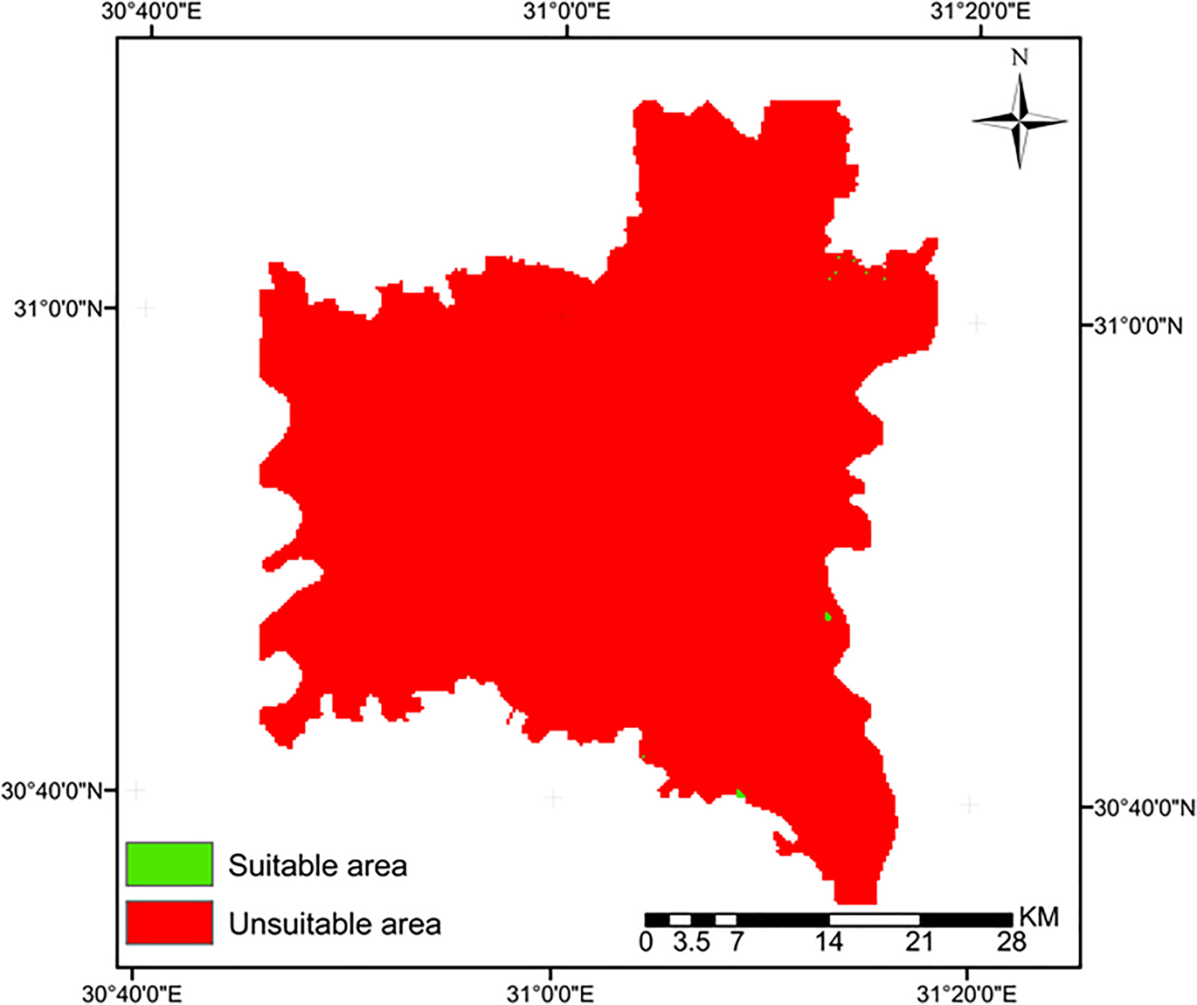
The final suitability map according to the Boolean method

Boolean methods are commonly employed during the initial screening phases to categorize areas into suitable and unsuitable categories (Malczewski, 1999). It is an accurate and simple method to determine landfill sites, particularly in cases where scaling weights within the overlay module is unnecessary (Delgado et al., 2008). The Boolean method can be used to select optimal locations exclusively through the use of criteria layers and restrictions. Initially, criteria were classified into suitable and unsuitable areas, as shown in Table 12. Subsequently, the Boolean tool in GIS was used to prepare the data, as presented in Fig. 6. Finally, the ultimate output suitability map was generated using fuzzy overlay approaches (Figs.7 and 8).

**Table 12:** The suitable limits for criteria to standardize maps (Boolean method)

Criteria	Acceptable standard for landfill site value selection
Depth to the groundwater	>7 m
Distance to urban centers	>5000 m
Distance to the surface water	>1000 m
Distance to the villages	>1000 m
Elevation	>4 m
Soil types	Silt clay
Slope	<10%
Distance to roads	>500 m
Land use	Unused land
Distance to the power lines	>300 m
Distance to railways	>500 m

### Weighted linear combination method (WLC)

The most popular approach for evaluating multi-scale assessments is the weighted linear combination (WLC) method (Barzehkar et al., 2019). The WLC method integrates the relative weights of the criteria (Tables 8, 10, and 11) along with the rating values (Fig. 5) to establish the final suitability maps. This is accomplished through three methods of MCDM (AHP, RSW, and SRS) using GIS, as shown in Fig. 7. In this process, a final value *A*_i_ is assigned to each location *i* using Eq. 10. Then, the *A*_i_ values are categorized into four different classes (suitable, moderately suitable, low suitable, and unsuitable) using the classification tool in GIS.

10 $${A}_{\mathrm{i}}=\sum\nolimits_{\mathrm{j}=1}^{\mathrm{n}}{W}_{\mathrm{j}}\ast {X}_{\mathrm{j}\mathrm{i}}$$

where *W*_j_ is the criterion weight of *j*, *X*_ji_ is the rating of criterion *j* at location *i*, and *n* is the criteria’s total number.

## Results and discussion

In this research, four techniques of MCDM were used: analytical hierarchy procedure (AHP), RSW, SRS, and the Boolean method. The weighted linear combination method (WLC) was utilized to integrate the relative weights of criteria derived from the three methods (AHP, RSW, and SRS) alongside the rating values to generate suitability maps for landfill site selection. Then, the suitability maps were categorized into four classes: suitable, moderately suitable, low suitable, and unsuitable. Ultimately, the final output for the suitable landfill site map was determined by combining the individual suitability maps from each method. This integration enables decision-makers to choose the optimal landfill site based on their preferences and requirements.

Initially, the Boolean method was utilized to determine appropriate sites for landfills in Al-Gharbia Governorate. The findings demonstrated that the suitable areas by this method are extremely limited, as shown in Fig. 8. Specifically, the percentage of land considered suitable amounts to a mere 0.03%. This area covers approximately 1 km^2^ and is scattered sparsely throughout Al-Gharbia Governorate where the largest part of this area measures 0.2 km^2^, rendering it inadequate for establishing landfill sites. Thus, the Boolean method proves excessively restrictive for the case study, a finding that is consistent with other published research. For instance, Delgado et al. (2008) found that 94% of the Cuitzeo Lake Basin in Mexico was considered inappropriate for municipal landfill use based on the Boolean method.

**Fig. 9:**
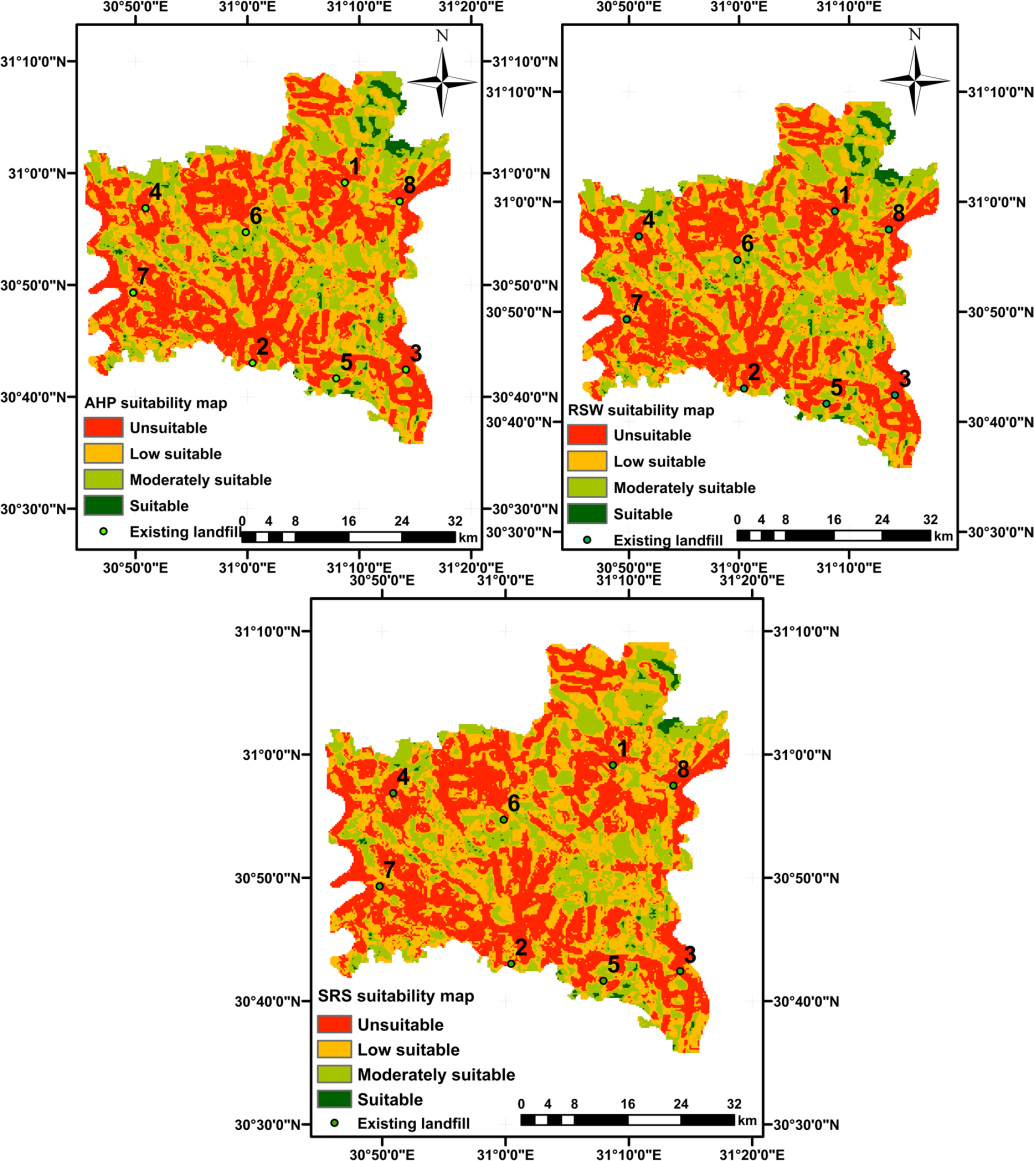
Suitability maps using three MCDM methods (AHP, RSW, and SRS)

The suitability maps for landfill sites in Al-Gharbia Governorate are presented in Fig. 9. The figure shows that the results of all three methods are quite comparable, especially between AHP and RSW. For the three methods, the areas for each categorized region are as follows: 796.22 km^2^ (41%) for the unsuitable area, 679.70 km^2^ (35%) for the low suitable area, 407.82 km^2^ (21%) for the moderately suitable area, and 58.26 km^2^ (3%) for the suitable area. The results reveal that a significant portion of the appropriate area (42.30 km^2^) is located in the northeastern part of Al-Gharbia Governorate, accounting for 72.6% of the total suitable area. The remaining suitable area is dispersed throughout the study region. In contrast, the unsuitable areas are concentrated in urban areas, while the low suitable areas are distributed sporadically across the study area and are adjacent to the unsuitable areas.

**Fig. 10:**
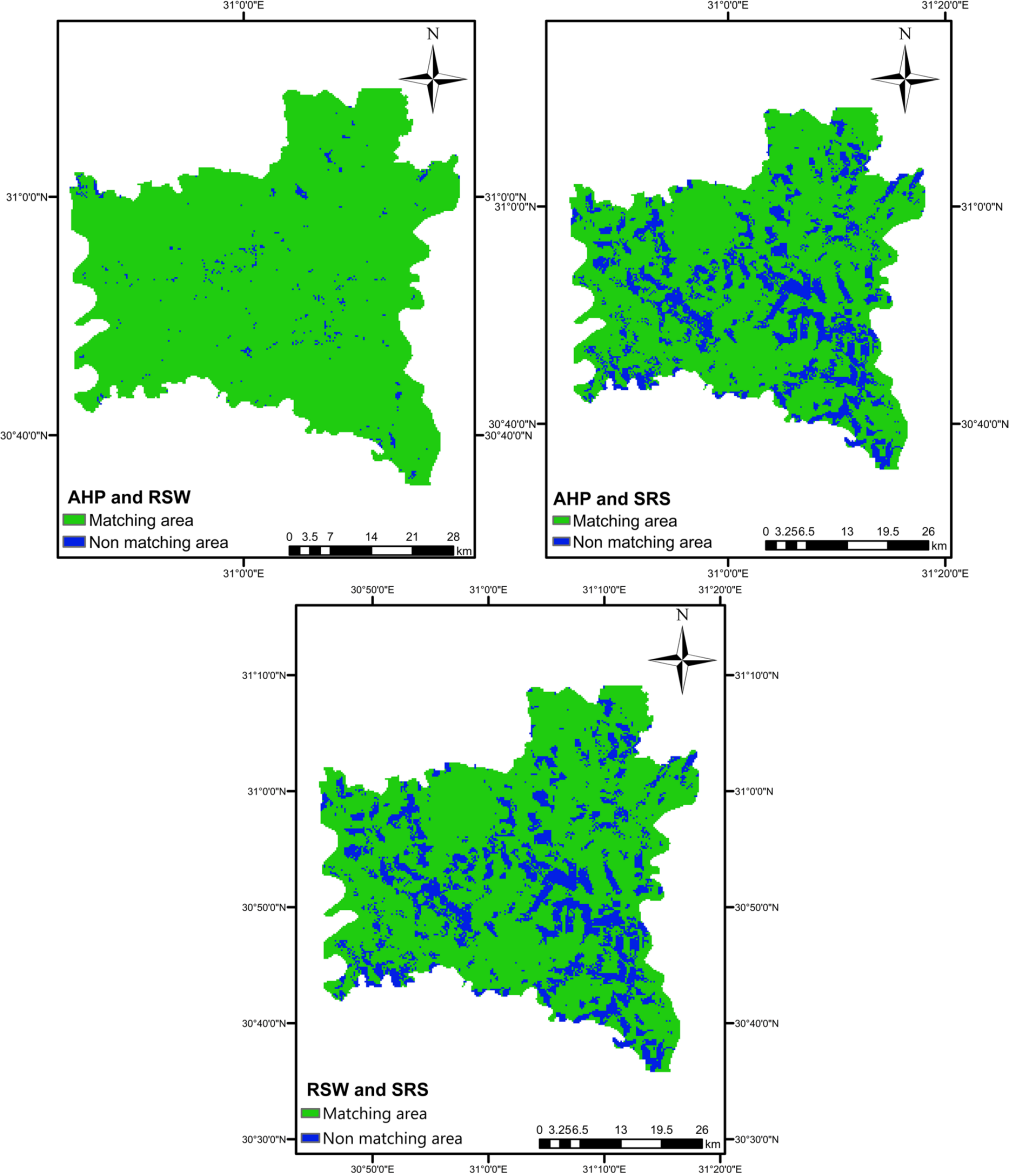
The matching maps of three methods of MCDM (AHP, RSW, and SRS) using change detection method

In this study, a change detection method was employed to individually compare two MCDM methods and differentiate between matching and non-matching pixels. When assessing AHP and RSW, it was found that the ratio of matching area is significantly high (98.4%), with very small non-matching area scattered across the case study. On the contrary, the comparison of the SRS method with the other two methods produced a matching area of 78.4% of the total area. Consequently, it can be concluded that the disparity in performance difference between AHP and RSW is minimal, while the difference between SRS and the other methods is substantial, as shown in Fig. 10.

**Fig. 11:**
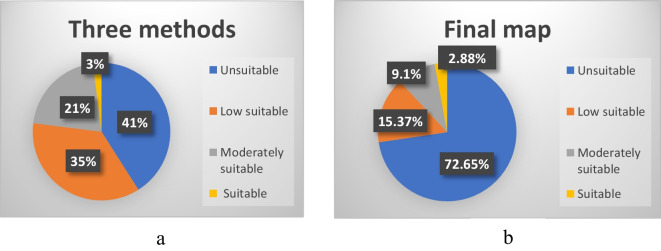
**a** The percentage of suitability area of three methods and **b** the percentage of suitability area of the final map

**Fig. 12:**
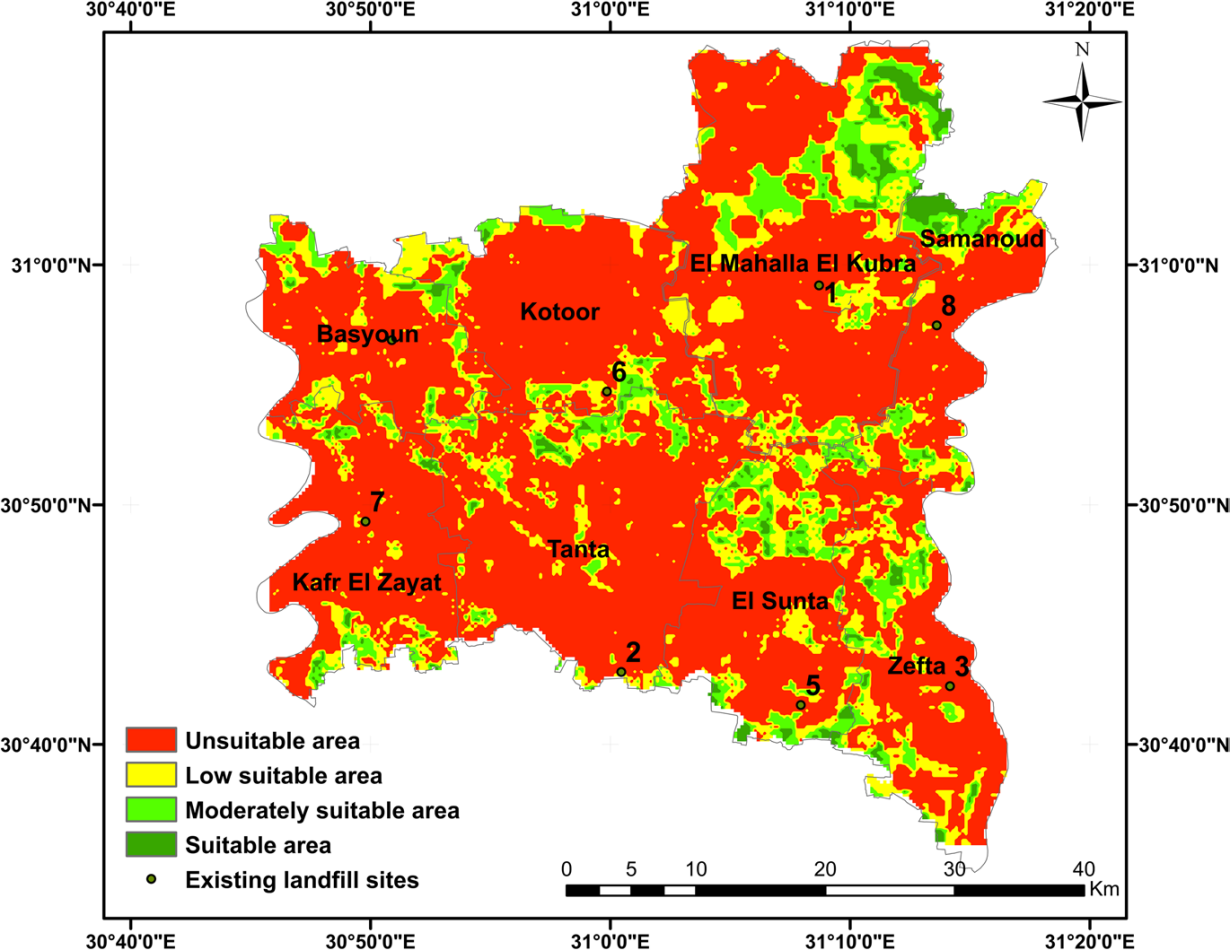
The final suitability map including the existing landfill sites

The final suitability landfill map was generated by combining the suitability maps produced using three methods of MCDM (AHP, RSW, and SRS) through the use of GIS. The combining method follows these interpretations: (1) if all the input values are deemed suitable, the output is deemed suitable; (2) if one or more inputs are unsuitable, the output is considered unsuitable; (3) if the inputs exhibit varying degrees of suitability, the least suitable option is chosen. The results show a disparity between the final suitability map and the three individual suitability maps, as illustrated in Fig. 11. In the final map, the suitable areas are slightly smaller in comparison to those in the individual suitability maps for each method, constituting 2.9% of the study area. These suitable areas are scattered throughout Al-Gharbia Governorate, as shown in Fig. 12. Particularly, the suitable areas are concentrated mainly in the northeast (Al-Mahalla), the central area (Tanta), and the northern sector (Kotour). On the other hand, the unsuitable areas in the final map encompass a large area (72.7%), compared to any of the individual suitability maps. The low suitable area and moderately suitable areas constitute a smaller fraction, representing 15.4% and 9.1%, respectively. These areas are primarily located in the northeast (Al-Mahalla), the central area (Tanta), and the southeast (Zefta).

In each center of Al-Gharbia Governorate, a site for landfill is situated, as shown in Table 13, which summarizes their main characteristics. In this study, the existing sites were validated using the final suitability map and the three individual suitability maps, as shown in Table 14. The results reveal that all the existing sites for landfill are located in unsuitable areas except for three sites (2, 5, and 6) which are located in low suitable areas but are in close proximity to unsuitable areas (Table 14 and Fig. 12). For example, site No. 3 is located in an unsuitable area according to all methods due to its close proximity to the urban center, surface water bodies, railways, power lines, and roads (Table 13). As a result, this site has direct adverse effects on the surrounding environment and local population. It leads to contamination of both surface and groundwater, contributes to road accidents, and results in unpleasant odors. Therefore, it is strongly recommended to either promptly close or relocate this site to a more suitable location. Four sites (1, 3, 7, and 8) are located within urban centers, a violation of global urban and environmental standards. Three sites (3, 7, and 8) are located very close to railways, with the distance between the railways and landfill sites being less than 50 m. Additionally, all sites are located very close to surface water bodies, and six sites (2, 3, 4, 5, 6, and 8) are close to villages. Regrettably, none of these existing sites meet basic scientific and environmental standards, thereby posing harm to both the environment and the local population.

**Table 13:** The main characteristics of the existing landfill sites in Al-Gharbia Governorate

Existing sites	Centers	C1 (m)	C2 (m)	C3 (m)	C4 (m)	C5 (m)	C6	C7	C8 (m)	C9	C10 (m)	C11 (m)
Site (1)	EL Mahala	5.2	0	50	2500	6	Silt clay	<10	30	Agriculture land	50	2700
Site (2)	El Sunta	4.7	4700	50	50	7	Silt clay	<10	100	Agriculture land	50	3500
Site (3)	Zefta	5.6	0	20	2000	7	Silt clay	<10	30	Agriculture land	50	30
Site (4)	Basyoun	7.20	1300	50	50	6	Silt clay	<10	500	Agriculture land	50	5000
Site (5)	El Sunta	6.5	3000	30	50	7	Silt clay	<10	900	Agriculture land	50	3300
Site (6)	Tanta	5.9	6000	30	30	7	Silt clay	<10	100	Agriculture land	500	1700
Site (7)	Kafr El Zayat	5.2	0	30	2500	8	Silt clay	<10	30	Agriculture land	50	50
Site (8)	Samanoud	6	0	100	1800	6	Silt clay	<10	930	Agriculture land	50	50

**Table 14:** Evaluating the existing landfill based on the final suitability map and the individual suitability maps of three methods of MCDM (AHP, RSW, and SRS)

Existing sites	AHP method	RSW method	SRS method	Final map
Site (1)	Unsuitable	Unsuitable	Unsuitable	Unsuitable
Site (2)	Low suitable	Low suitable	Low suitable	Low suitable
Site (3)	Unsuitable	Unsuitable	Unsuitable	Unsuitable
Site (4)	Unsuitable	Unsuitable	Unsuitable	Unsuitable
Site (5)	Low suitable	Low suitable	Low suitable	Low suitable
Site (6)	Low suitable	Low suitable	Low suitable	Low suitable
Site (7)	Unsuitable	Unsuitable	Unsuitable	Unsuitable
Site (8)	Unsuitable	Low suitable	Unsuitable	Unsuitable

**Fig. 13:**
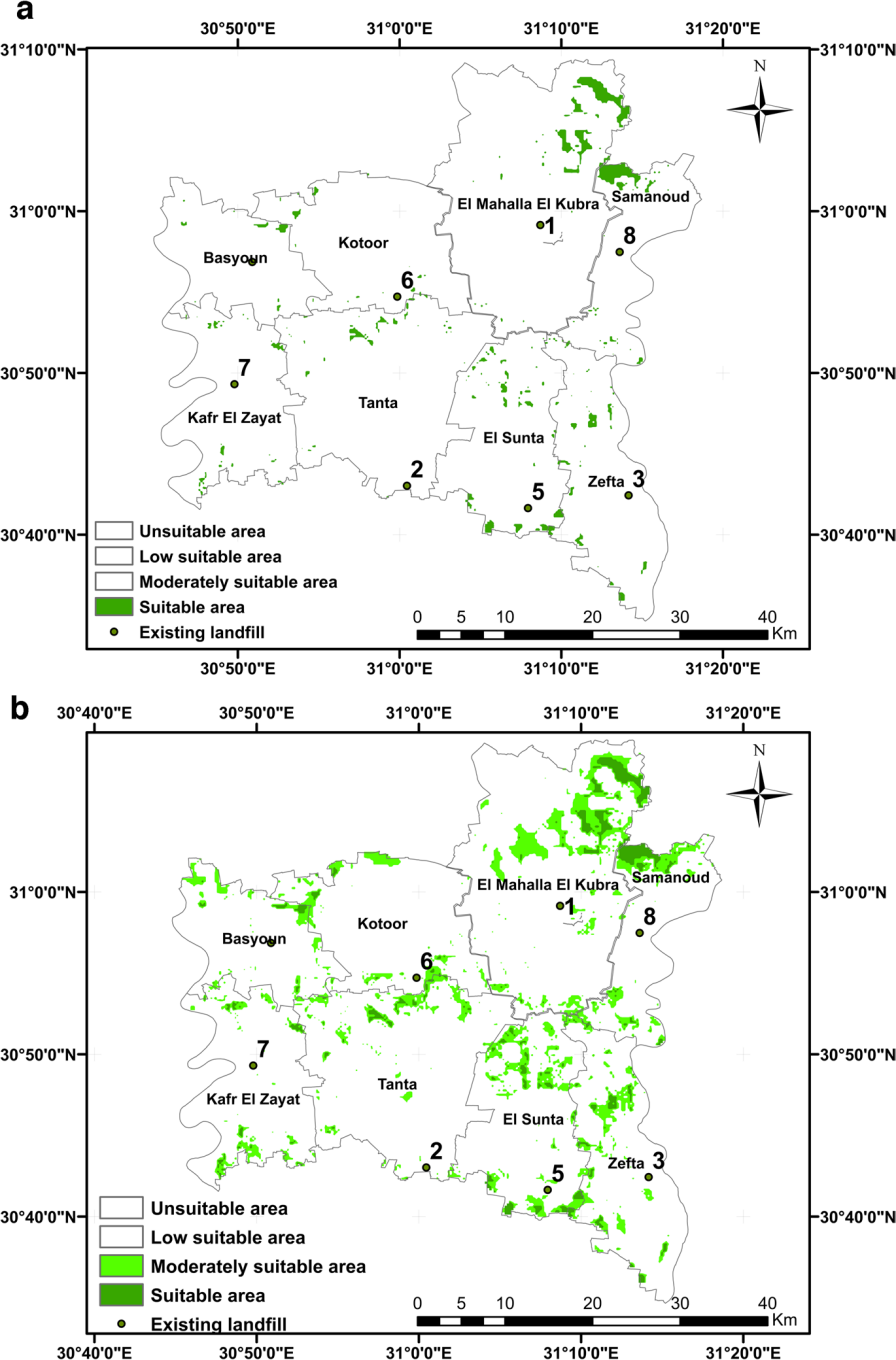
The final suitability map for EL-Gharbia Governorate centers including the existing landfill sites: **a** suitable, **b** moderately suitable

Figure 13 illustrates the final landfill suitability map for Al-Gharbia Governorate encompassing the eight centers: Basyoun, Koutor, El Mahla El Kouba, Samanoud, Kafr El Zaiat, Tanta, El Santa, and Zefta. The figure displays the suitable landfill sites based on the combination of the three AHP, RSW, and SRS approaches. In addition, the figure introduces areas classified as moderately suitable and suitable, both determined using the same three MCDM methods. The outcomes confirmed that all existing sites are situated outside the regions categorized as moderately suitable and suitable. The spatial distribution shown in the figure underscores that the suitable areas are distributed across the various centers within El-Gharbia. Most of the suitable areas are located in the central part of the governorate, particularly in Tanta, El Santa, and Zefta. In addition, suitable areas are also identified in the northern and northeastern sectors, covering El Mahla El Koubra and Samanoud centers. The final spatial landfill suitability map for Al-Gharbia Governorate holds potential for practical implementation in waste management, particularly within the central area of the Nile Delta, Egypt.

## Conclusion

The aim of this current research is to identify appropriate landfill sites in Al-Gharbia Governorate and to validate existing landfill sites. Eleven key criteria were considered, and they were categorized into two groups: natural criteria (groundwater, surface water, soils, elevation, slope, and land use) and artificial criteria (roads, railways, urban areas, villages, and power lines). Each criterion was subdivided into sub-criteria, and a rating value ranging from 0 to 10 was assigned to each. These ratings were established using information from published studies and recommendations from scientific experts. Four techniques of multi-criteria decision-making (MCDM) were used: AHP, RSW, SRS, and the Boolean method. The resulting suitability maps were divided into four categories: suitable, moderately suitable, low suitable, and unsuitable. The final suitability map was generated by integrating the three individual suitability maps produced by the different methods, using the WLC technique. Furthermore, the existing landfill sites within Al-Gharbia Governorate were assessed based on the resulting suitability maps. The following conclusions can be drawn based on the current research:

1. The application of the Boolean method results in a very limited proportion of suitable areas for landfill creation within Al-Gharbia Governorate (0.03%). Consequently, the Boolean method proves to be highly restrictive for the study area.
2. The outcomes obtained from the three methods (AHP, RSW, and SRS) concerning landfill site selection within Al-Gharbia Governorate exhibit notable similarity, particularly between AHP and RSW. The distribution across different suitability categories is as follows: 41% for unsuitable areas, 35% for areas of low suitability, 21% for moderately suitable areas, and 3% for areas deemed suitable.
3. The final suitability map resulting from the integration of the three MCDM methods reveals a substantial disparity when compared to the individual suitability maps. In the final map, the unsuitable areas encompass a significant expanse (72.7%), which is notably larger compared to any of the individual suitability maps (41%). The areas classified as having low suitability and moderate suitability are relatively small, constituting 15.4% and 9.1% of the total area, respectively. These regions are situated in the northeastern part (Al-Mahalla), the central area (Tanta), and the southeastern part (Zefta).
4. While suitable areas are distributed across various locations within Al-Gharbia Governorate, substantial portions of them are concentrated primarily in the northeastern region (Al-Mahalla), the central area (Tanta), and the northern area (Kotour).
5. All the existing landfill sites are situated within areas classified as unsuitable or having low suitability. This is largely due to their proximity to, and in some cases even being within, urban centers, villages, surface water bodies, railways, power lines, and roads.

The significant proportion of unsuitable areas in the study region can be attributed to the agricultural nature of the governorate, its high population density, extensive surface water coverage, elevated groundwater levels, and extensive road and electricity networks. Presently, all landfill sites in Al-Gharbia Governorate fail to meet basic scientific and environmental standards, leading to severe repercussions on the surrounding environment, public health, groundwater integrity, and agricultural lands. Consequently, a strong recommendation for upcoming research involves conducting comprehensive environmental studies on the existing landfill sites. The findings of such studies could then guide decision-makers in the effective management of municipal solid waste in the central area of the Nile Delta, specifically within Al-Gharbia Governorate, Egypt. The methodology used in this research to identify suitable landfill sites is founded upon the selected criteria and the availability of data. Therefore, it is advisable for future research efforts to incorporate more up-to-date and extensive data, particularly concerning groundwater conditions. Furthermore, exploring suitable landfill sites within other analogous regions is highly recommended.

## Data Availability

The data is available on request to the corresponding author.
